# Postpartum Hypernatremia with Extrapontine Rhabdomyelinolysis: A Case Report

**DOI:** 10.5339/qmj.2022.45

**Published:** 2022-10-17

**Authors:** Garima Choudhary, Faisal Qureshi, Anka Arora, Nikhil Kothari, Sarbesh Tiwari, Pradeep Bhatia

**Affiliations:** ^1^Department of Anaesthesiology & Critical Care, AIIMS Jodhpur E-mail: faisal.qur.90@gmail.com; ^2^Senior Resident, Department of Neurology, AIIMS Jodhpur; ^3^Department of Interventional Radiology, AIIMS Jodhpur

**Keywords:** Postpartum, pregnancy, extrapontine, osmotic demyelination syndrome, hypernatremia, flaccid paralysis

## Abstract

Hypernatremia (serum sodium>160 meq/L) present with diverse neurological manifestations, ranging from flaccid paralysis to impaired cognition, encephalopathy, and even deep coma. Osmotic demyelination refers to changes in brain cells because of an acute change in plasma osmolality. It is further divided into two types, i.e., central pontine myelinolysis (CPM) and extrapontine myelinolysis (EPM). Patients with EPM, besides spasticity, may also present with other movement disorders such as catatonia, parkinsonism, and dystonia. We present a case of a postpartum woman bought to the emergency department by her relatives in an unconscious state. In view of poor sensorium (Glasgow coma scale < 7), she was intubated and received mechanical ventilatory support. On admission, computed tomography ofthebrain was normal, and the patient was transferred to the intensive care unit (ICU) for further management. The preliminary work-up in the ICU showed hypernatremia (serum sodium of 182 mEq/L) with hyper-osmolality (359 mOsm/kgH_2_O). She was managed as per the ICU protocol for hypernatremia. During her ICU stay, her sensorium improved, but she developed flaccid paralysis and was quadriplegic. Thus, a tracheostomy was performed, and she was weaned from the ventilator. After ventilator weaning, she was transferred to the ward for further rehabilitation. During rehabilitation, the patient was able to sit and takefoodorally.To date, only a few cases are reported in postpartum women with acute severe hypernatremia caused by high-grade fever and loss of body fluids leading to extra-pontine demyelination and flaccid paralysis. This case highlightsthat prompt recognition and appropriate intervention can improve the outcomes in these patients.

## Introduction

Hypernatremia due to various conditions presents with diverse neurological manifestations, ranging from flaccid paralysis to impaired cognition, encephalopathy, and even deep coma. Rapid correction of serum sodium levels leads to central pontine myelinolysis (CPM) and extrapontine myelinolysis (EPM), which can be associated with encephalopathy and other neurological complications. The normal serum sodium level is between 135 and 145 mEq/L. Serum sodium levels of >160 mEq/L are defined as severe hypernatremia. Osmotic demyelination refers to changes in brain cells because of acute change in plasma osmolality and subsequent failure of brain cells to adapt to these changes in serum osmolality.^1^ It is further divided into CPM and EPM. However, the underlying pathology is similar in both diseases because of the involvement of the pons, but they differ in clinical manifestations. CPM presents initially with encephalopathy or seizures, followed by flaccid quadriparesis. It ultimately leads to spastic rigidity in all four limbs, which may or may not be associated with dysarthria and dysphagia. In patients with EPM, besides spasticity, other movement disorders such as catatonia, parkinsonism, and dystonia may be seen.^2^ Overall, osmotic demyelination syndrome (ODS) occurs in the range of 0.4% to 0.56% in all patients with neurological problems admitted to tertiary hospitals. The introduction of magnetic resonance imaging (MRI) has led to an increased incidence of 0.3%–1.1%.^3,4^ However, there is no data about the difference in ODS in postpartum patientswhen compared with the normal population. To date, very few casesofextrapontine demyelination in postpartum patients due to hypernatremiaare reported.^5-7^ Herein, we report a case of postpartum hypernatremia leading to EPM.

## Case Report

A 29-year-old woman, G2P1A0, was brought to the emergency department (ED) in an unconscious state. She had a history of fever and decreased urine output for 4–5 days. She also had a recent history of a full-term normal vaginal delivery 9days before at a local hospital without any antenatal or intranatal complications. She did not receive any antibiotics during labor, and bleeding during delivery was approximately 500 mL. No partogram was available in the documents, and she had no history of postpartum hemorrhage. Her relatives explained that 4 days after the delivery, she started having episodes of fever (37.7°C–38.3°C) with loose stools (4–5 episodes per day). Subsequently, she was admitted to a government medical college and hospital where she delivered vaginally. She received paracetamol and fluids intravenously(the positive balance at the end of 2 days was 1500 ml). No other obstetrical postpartum complication, such as excessive bleeding, breast mastitis, or lactation problems, occurred. She was discharged home after 2 days of hospitalization, as her fever subsided, andresults of relevant tests such as complete blood count, renal function test, liver function test, and coagulation parameters were normal. She has advised tablet paracetamol and multivitamin syrup on discharge. However, on day 9 after the delivery, she was bought to the ED in an unconscious state, with complaints of fever and irrelevant talk for 1 day. According to her relatives, shehad excessive sweating and decreased intake of water in the last 2 days. There was no accurate record of fluid intake between days 4 and 8, but it was generally reduced because of the custom in thepatient's community.

On examination in the ED, her pupils were normal in size and reacting to light. Neck rigidity and limb hypotonicity were not observed, and her plantar response was flexor. She responded to painful stimuli by opening up her eyes, without a verbal response, and normally flexing her limbs. Her Glasgow coma scale (GCS) score was 7 (E2V1M4). She had a fever of 104.5°F (40.2°C), and her blood pressure was 100/68 mmHg, without any vasopressors, with a pulse rate of 144 beats per minute. She was intubated based on GCS of 7 and was placed on mechanical ventilation in the ED. Her provisional diagnosis was meningoencephalitis with a differential of tropical fever, and she was transferred shifted to the ICU for further management. During the first hospitalization, she did not receive any antibiotics, so we gave her ceftriaxone 2 gintravenously twice a day based on the provisional diagnosis.

**Figure 1.**
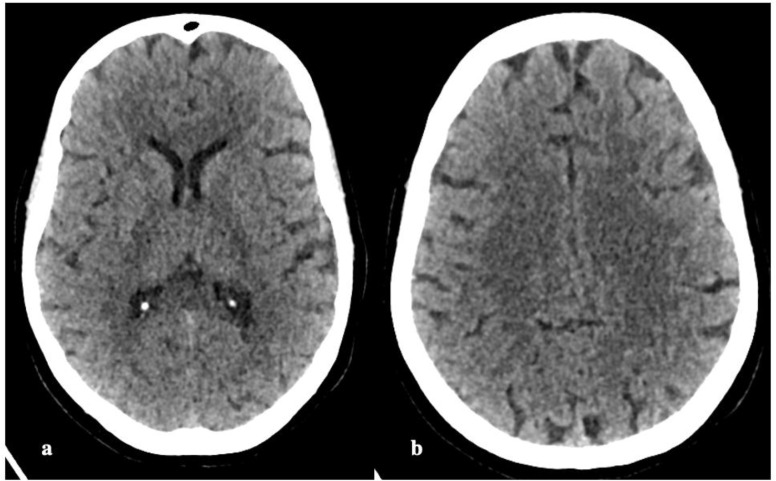
Non-contrast computed tomography of the head at the level of the basal ganglia (a) and centrum semiovale (b) does not show any obvious abnormalities.

Her CT of the brain was normal (Figure 1), following which a cerebrospinal fluid (CSF) was sent for examination. The laboratory evaluation showed severe hypernatremia with serum sodium (Na^+^) level of 182meq/L, chloride level of 148 mEq/L, plasma osmolality of 439 mOsmol/kg, urine osmolality of 782 mOsmol/kg, hemoglobin of 12.7 g/dL, hematocritof 47.8%, total white blood cell count of 16.68 ×  10^3^/μL, platelet count of 132 × 10^3^/μL, blood urea of 171 mg/dL, and serum creatinine of 2.56 mg/dL. Other investigations including liver function test, coagulation parameters, and CSF biochemical report and cell counts were normal. All cultures were sterile. Dengue antibody and malaria rapid antigen were also negative.

**Figure 2.**
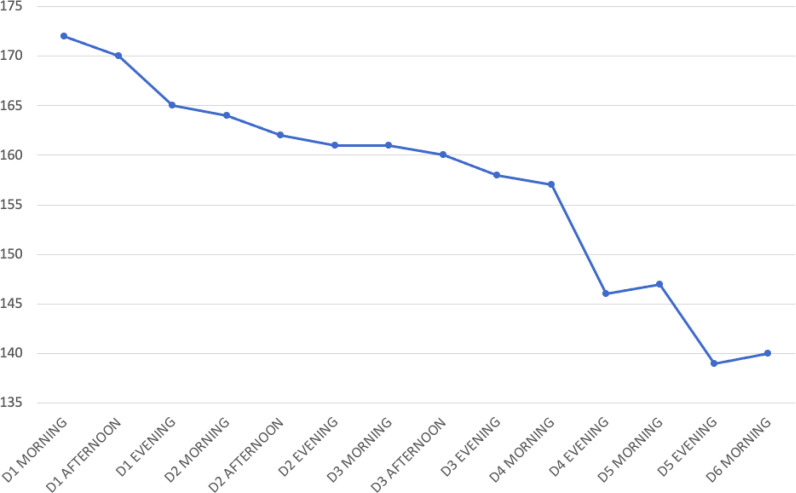
Change in serum sodium (mEq/L) over a few days (D).

**Figure 3.**
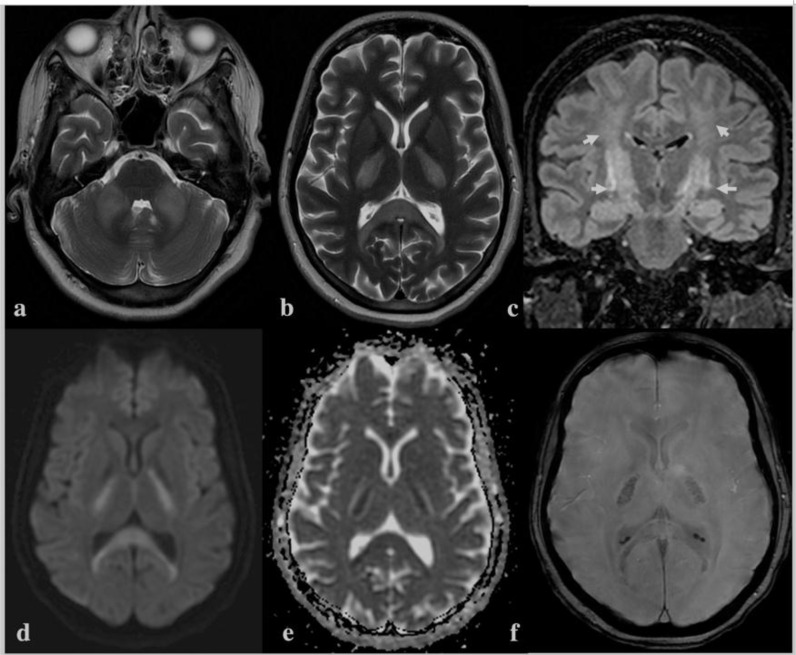
Axial tbl2 (a, b) images show abnormal hyperintense signal involving bilateral middle cerebellar peduncles, splenium of the corpus callosum, and posterior limb of the internal capsule. The coronal fluid-attenuated inversion recoveryimage (c) demonstrated selective involvement of the corticospinal tract (short arrows) giving a “wine-glass” appearance. The involved areas show restriction on diffusion-weighted images (d,e) with evidence of microhemorrhages on the susceptibility-weighted images (f). The imaging features are characteristic of hypernatremic encephalopathy with osmotic myelinolysis and rhabdomyolysis

Ultrasound screening of inferior vena cava showed collapsibility of >50%. Fluid resuscitation by intravenous administration of balanced crystalloids was started. A nasogastric tube was inserted, and free water replacement was started as the total free water deficit was calculated to be approximately 7.8 L. Nasogastric feeding was also started as per the ICU protocol, and glycemic control was maintained. Serial monitoring of serum sodium was conducted using arterial blood gas analysis (Figure 2). The rate of sodium correction was kept below 1 mEq/L/h, and her urine output was monitored (50–70 mL/h). She was kept on a ventilator, and her neurological status was assessed dailyduring ICU rounds. Over the next days, her serum sodium level dropped to 142 mEq/L, and her sensorium also improved. Sedation was stopped, and weaning trials were attempted. She could follow verbal commands by opening and closing her eyes and could identify her relatives by nodding her head. However, she was unable to move her limbs, and her power grade was between 1/5 to 2/5 in all four limbs. There was drooling of secretions from the angle of the mouth, which was suggestive of bulbar weakness. In view of her poor muscle power, neurology consultation was taken, and MRIof the brain and spine revealed symmetrical tbl2, and fluid-attenuated inversion recovery revealed hyperintense signal and restricted diffusion along the corticospinal tract involving the centrum semiovale, corona radiata, posterior limb of the internal capsule, and crus cerebri, appearing as “wine-glass” on the coronal image. Symmetrical signal changes also demonstrated involvement of the middle cerebellar peduncle and splenium of the corpus callosum. The gradient images showed microhemorrhages in the involved region. The imaging features were suggestive of postpartum hypernatremic encephalopathy with osmotic extrapontine myelinolysis (Figure 3). Following the MRI report, measurement of serum creatinine phosphokinase was advised, which was 475 IU, and her serum lactic dehydrogenase was 1229 IU/L. Her urine myoglobin was 1200 ng/mL. A final diagnosis of postpartum hypernatremic encephalopathy with osmotic EPM and rhabdomyolysis was made. MRI remains to be the chief diagnostic tool for ODS and EPM.^3^ Her rhabdomyolysis resolved in a week with judicious fluid management. Considering bulbar involvement, it was difficult to extubate her, so percutaneous tracheostomy was performed on day 12 in the ICU. She was weaned from the ventilator after the PCT. As she was unable to swallow semisolids, gastroenterologists for advised percutaneous endoscopic gastrostomy for long-term feeding, which was performed by the gastroenterologists on the next day.

Her rehabilitation was started in the ICU. She was able to sit with support, and her power improved to grade 3/5 in the upper limbs and grade 2/5 in the lower limbs. She was then transferred to the ward for long-term rehabilitation under the follow-up of the department of physical medicine and rehabilitation.

## Discussion

Hypernatremia occurs mostly because of a disturbance of water balance in the body involving excessive water loss due to sweating, diuresis, vomiting, or deficiency of antidiuretic hormone (ADH). It rarely occurs in the postpartum periodwhen the patient has a good hydration status. In the presented patient, the hypernatremia was possibly caused by dehydration because of poor water intake and fever. A few cases of hypernatremia in the postpartum period have been reported, although the exact etiological factor is not known as published by Shrier et al.; during late pregnancy, there is a three- to four-fold increase in plasma vasopressinase levels, secreted by placental trophoblasts. This enzyme rapidly metabolizes the plasma vasopressin, resulting in a partial decrease in ADH secretion during the immediate postpartum period and an increase in serum sodium levels.^8^

According to Naik et al. regarding the seasonal postpartum hypernatremic encephalopathy with osmotic demyelination, our patient hadencephalopathy but with extrapontine neurological symptoms and rhabdomyolysis. In previously reported cases, 9 of 11 developed quadriparesis, and eight patients had involvement of corticospinal and corticobulbar tracts, whereas four developed seizures.^9^

A sudden change in serum sodium levels (i.e., >12 mEq/day) may affect brain functions, and pontine myelinolysis can occur resulting in comatose, but cases of extrapontine demyelination with flaccid paralysis and rhabdomyolysis are rarely reported from this part of the world.^10,11^ Yamada et al. concluded that high serum sodium levels result in osmotic demyelinating syndrome and even gradual correction of serum sodium levels would not help improve the neurological status.^12^ Kuruvilla et al. reported MRIfindings of bilateral symmetrical hyperintensities in the extrapontine region (wine-glass sign) in primary lateral sclerosis.^13^ Similar MRI findingswere observed in this patient, which were also suggestive of EPM in this case.

## Conclusion

A postpartum patient with sudden fluid shifts may present in an unresponsive state with high serum sodium levels and rhabdomyolysis. These states should not be always considered central pontine demyelination. These patients may have extrapontine demyelination and may become responsive after 48–72 hwith a decreased limb power, which gradually recovers.

## Ethics

Informed consent was obtained from the patient's relative.
